# Association of type 2 diabetes mellitus with postoperative outcomes following single-level endoscopic lumbar interbody fusion through a posterior approach: A global propensity score–matched cohort study

**DOI:** 10.1371/journal.pone.0358422

**Published:** 2026-09-18

**Authors:** Phuoc Giau Dang, Yu-Pin Chen, Tan Thanh Nguyen, Dat Huu Nguyen, Yi-Jie Kuo

**Affiliations:** 1 The International Ph.D. Program in Medicine, College of Medicine, Taipei Medical University, Taipei, Taiwan; 2 Department of Orthopedics, Faculty of Medicine, Can Tho University of Medicine and Pharmacy, Can Tho, Vietnam; 3 Department of Orthopedics, Wan Fang Hospital, Taipei Medical University, Taipei, Taiwan; 4 Department of Orthopedic Surgery, School of Medicine, College of Medicine, Taipei Medical University, Taipei, Taiwan; 5 Department of Orthopedic Surgery, Hsin Kuo Min Hospital, Taipei Medical University, Taoyuan City, Taiwan; Kyung Hee University Hospital at Gangdong, Kyung Hee University College of Medicine, KOREA, REPUBLIC OF

## Abstract

**Background:**

Type 2 diabetes mellitus (T2DM) has been associated with impaired bone healing and may increase the risk of pseudarthrosis after lumbar fusion. Its impact after endoscopic lumbar interbody fusion (Endo-LIF) performed through a posterior approach remains unclear.

**Methods:**

In this retrospective cohort study using the TriNetX Global Collaborative Network, adults undergoing single-level Endo-LIF through a posterior approach between 2015 and 2025 were identified from ICD-10-PCS procedure codes, which record a percutaneous endoscopic interbody fusion approached from behind but do not distinguish uniportal from biportal technique or posterior from transforaminal variants. Patients with and without T2DM were propensity score–matched 1:1 on demographic and clinical variables. The primary outcome was code-defined pseudarthrosis (ICD-10-CM M96.0) within three years; secondary outcomes were 90-day complications and one-year healthcare utilization. Time-to-event outcomes were analyzed with Kaplan–Meier estimates, log-rank tests and Cox regression.

**Results:**

After matching, 755 patients remained per cohort. Early complications and healthcare utilization were comparable (all p > 0.05). Code-defined pseudarthrosis was recorded more often with T2DM at every interval (observed proportions 11.5% vs 6.2% by 36 months; all p < 0.01). Kaplan–Meier cumulative incidence at 36 months was 12.0% (95% CI 9.8–14.6) with T2DM versus 6.4% (95% CI 4.9–8.5) without (log-rank p = 0.0003), an absolute risk difference of 5.5% (95% CI 2.6% to 8.5%); the hazard ratio was 1.90 (95% CI 1.33–2.71).

**Conclusion:**

In this database cohort, T2DM was associated with a higher rate of code-defined pseudarthrosis after single-level Endo-LIF, despite similar short-term outcomes. Because the outcome was code-defined and the procedure codes do not characterize operative technique, these findings describe an association only: they do not establish that T2DM independently causes radiographic nonunion, and are consistent with, but do not demonstrate, a contribution of systemic metabolic factors to long-term fusion.

## Introduction

Among metabolic diseases, type 2 diabetes mellitus (T2DM) ranks as one of the most widespread worldwide, currently influencing roughly one in ten adults and an estimated 828 million people [1–3]. Its complications extend beyond glucose metabolism and include detrimental effects on bone quality, angiogenesis, and delaying wound healing [4–7]. Sustained hyperglycemia facilitates the accumulation of advanced glycation end-products, inhibits osteoblast function, and induces microvascular impairment, thereby delaying bone regeneration and reducing fusion stability following spinal procedures [8,9]. Consequently, the diabetic cohort showed an increased incidence of surgical complications, including delayed union and pseudarthrosis, accompanied by higher overall healthcare costs driven by prolonged inpatient care after spinal fusion [10–12]. As the prevalence of both diabetes and spinal degenerative diseases continues to rise [13,14], understanding the interaction between these conditions has become increasingly relevant to surgical decision-making and postoperative management.

Most prior research has focused on the association between diabetes and fusion outcomes in open and minimally invasive lumbar fusion surgery [15,16]. These studies consistently show that T2DM is associated with inferior fusion rates and higher reoperation risk [17,18]. However, its influence on endoscopic lumbar interbody fusion (Endo-LIF) performed through a posterior approach has not been clearly defined. Throughout this report Endo-LIF refers to single-level procedures recorded as percutaneous endoscopic interbody fusion approached from behind, and is used as a descriptive label for the operation as recorded in administrative coding rather than as a designation of a specific endoscopic technique. In contrast to open or minimally invasive approaches, Endo-LIF provides direct endoscopic visualization with minimal soft-tissue trauma and smaller incisions. These features support a faster recovery and reflect the current trend toward less invasive, anatomy-preserving spine surgery [19–21]. Whether T2DM is associated with poorer long-term fusion outcomes among patients undergoing this operation has not been examined.

This retrospective cohort analysis explored the association between T2DM and outcomes after single-level Endo-LIF, with attention to perioperative complications, medical resource use, and fusion success. Clarifying this association may help identify at-risk populations and guide perioperative management strategies.

## Materials and methods

### Study design and data source

Data for this retrospective cohort analysis were obtained from the TriNetX Global Collaborative Network, which compiles de-identified electronic medical records contributed by over 150 healthcare institutions worldwide. The database contains longitudinal information on demographics, diagnoses, medical and surgical procedures, laboratory findings, and clinical outcomes, encompassing more than 150 million patients. Diagnostic and procedural data were classified according to the International Classification of Diseases, 10th Revision, Clinical Modification and Procedure Coding System (ICD-10-CM/PCS) [22]. Data were accessed on July 7, 2025.

All information within TriNetX is de-identified in compliance with the Health Insurance Portability and Accountability Act (HIPAA) and relevant privacy regulations. The TriNetX platform ensures that all patient data are fully anonymized and comply with HIPAA privacy standards. The authors did not have access to any information that could identify individual participants at any stage of the study.

The study protocol was reviewed and approved by the Institutional Review Board of Changhua Christian Hospital (CCH IRB No. 250612). The requirement for informed consent was waived due to the use of de-identified data.

### Patient selection

Adult patients who underwent single-level Endo-LIF between January 1, 2015, and January 1, 2025, were identified within the TriNetX Global Collaborative Network (n = 15,994). Patient identification was performed using ICD-10-PCS procedure codes for single-level percutaneous endoscopic lumbar interbody fusion performed through a posterior approach (0SG047J, 0SG04KJ, 0SG04JJ, 0SG04AJ, and 0SG04ZJ). These codes identify the cohort by coded operative approach only: they do not distinguish uniportal full-endoscopic from biportal endoscopic technique, nor a posterior interbody fusion from a transforaminal endoscopic variant, and Endo-LIF is therefore used throughout as a code-defined descriptor rather than a technical designation.

Patients with T2DM were defined as those who met both of the following criteria:

(a) presence of an ICD-10-CM diagnostic code for T2DM (E11) to exclude other types of diabetes (E08, E09, E10, E12, and E13), and
(b) a documented HbA1c ≥ 6.5% recorded at any time prior to surgery.

This combined definition was applied to ensure diagnostic accuracy and consistency with the American Diabetes Association (ADA) Standards of Medical Care in Diabetes 2025 [23].

**Fig 1:**
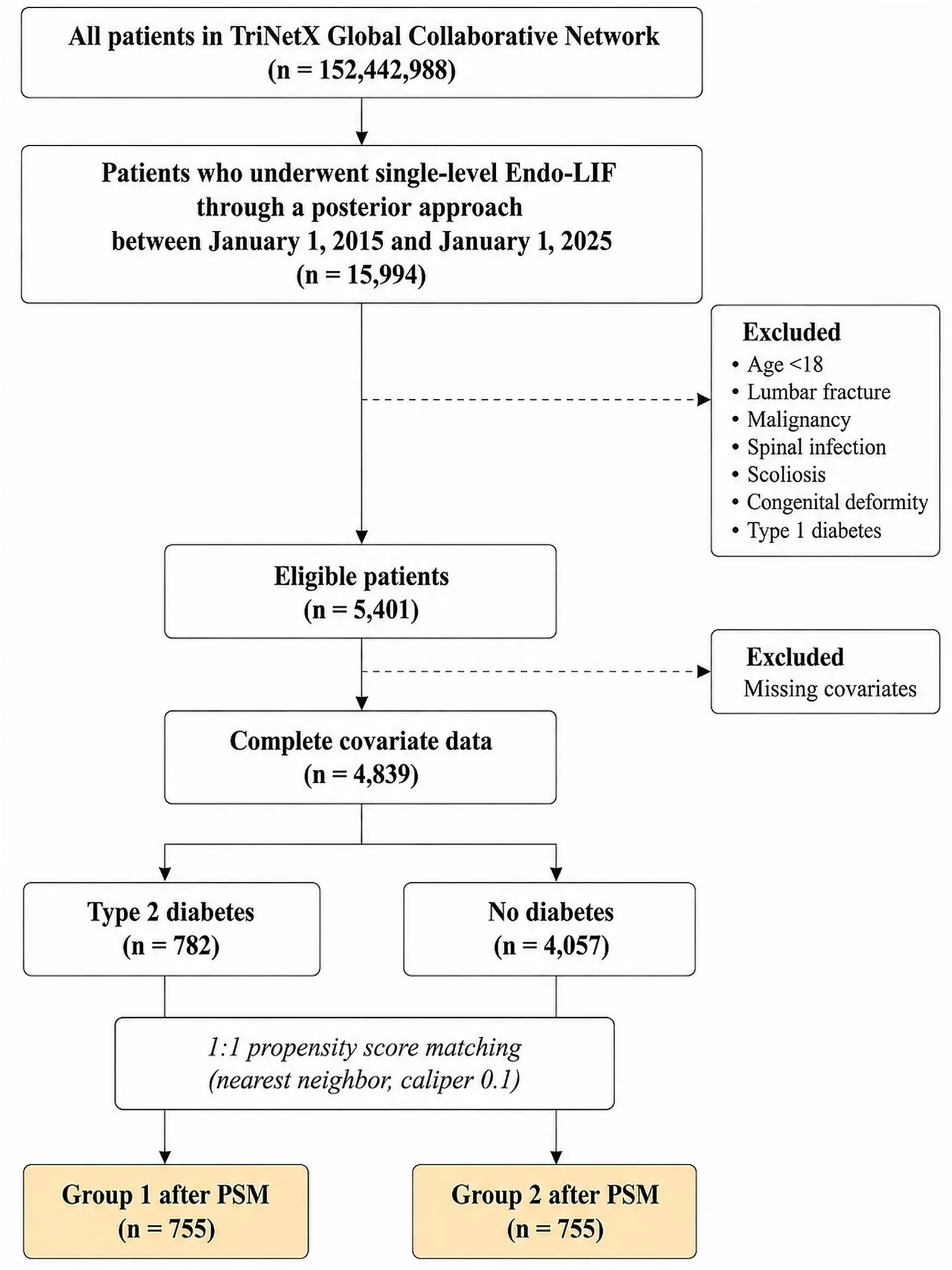
Flowchart patient selection.

Patients were excluded if they were younger than 18 years or had diagnostic codes indicating lumbar fracture (S32), malignant bone tumor (C41), scoliosis (M41), congenital spinal deformity (Q76), spinal infection and inflammatory spondylopathy (M46), or type 1 diabetes (E10). After the inclusion and exclusion criteria were applied, the database query returned 5,401 patients (894 with T2DM and 4,507 without diabetes). Propensity-score matching on the TriNetX platform is performed on complete cases; 562 patients (112 with T2DM and 450 without diabetes) lacked a recorded value for at least one matching covariate, were therefore not eligible for matching, and were excluded at this step. The analytic cohort comprised 4,839 patients, of whom 782 had T2DM and 4,057 had no diabetes. Propensity-score matching (PSM) at a 1:1 ratio yielded two cohorts of 755 patients each, which were used for the final comparison (Fig 1).

### Covariates and matching

The baseline variables considered in the propensity-score model comprised demographic factors (age, sex, race, and ethnicity), smoking history, and major comorbidities including hypertension, hyperlipidemia, osteoarthritis, ischemic heart disease, obesity, nicotine dependence, chronic kidney disease, and osteoporosis. Propensity scores were generated through logistic regression, and a 1:1 matching process was conducted using a nearest-neighbor method with a caliper of 0.1 standard deviations of the logit score [24,25].

The degree of covariate balance after matching was assessed based on standardized mean differences (SMDs), where an SMD below 0.10 denoted satisfactory equivalence between groups. All baseline variables met this criterion following matching.

### Outcomes and follow-up

Early postoperative events were recorded for 90 days and included pneumonia, urinary tract infection, anemia, the need for blood transfusion, and pressure ulcer. Measures of healthcare use, such as emergency department visits and unplanned readmissions, were examined during the first postoperative year.

Code-defined pseudarthrosis served as the primary outcome. It was identified using ICD-10-CM code M96.0 (pseudarthrosis after fusion or arthrodesis); revision or reoperation procedure codes were not used to define the outcome. Patients were followed from the index procedure until code-defined pseudarthrosis, their last recorded encounter, or the end of the 36-month window, whichever came first, and cumulative incidence was estimated over this period. Because the index window extended to January 1, 2025 while the data were accessed on July 7, 2025, patients treated late in the study period could not contribute a full 36 months of potential follow-up; observed proportions are therefore reported alongside Kaplan–Meier estimates that account for censoring.

### Statistical analysis

Covariate balance was summarized using standardized mean differences. Pre-matching comparisons used the chi-square test for categorical variables and independent t-tests for continuous variables. In the matched cohorts, the two groups were compared as independent samples using the analytic tools available on the platform: categorical outcomes were compared with the chi-square test and summarized as risk ratios with 95% confidence intervals, and time-to-event outcomes were analyzed with a Cox proportional hazards model together with Kaplan–Meier estimates and the log-rank test. The platform does not provide conditional or robust-variance methods and does not permit patient-level export, so the matched-pair structure could not be modeled directly; in propensity-score matched samples, analyses that ignore the pairing generally yield conservative variance estimates [26]. The proportional hazards assumption was tested using the proportionality test based on scaled Schoenfeld residuals implemented in the platform [27], and was additionally assessed graphically with a complementary log–log plot (S1 Fig). Cumulative incidence of code-defined pseudarthrosis was estimated by the Kaplan–Meier method at 6, 12, 24 and 36 months. The platform reports each survival estimate together with its 95% confidence interval, derived on the complementary log–log scale; the standard error of each estimate was recovered from these reported confidence limits rather than computed from risk-set counts, which the platform does not export. The absolute risk difference between cohorts at each time point, and its confidence interval, were calculated from the unrounded censoring-adjusted estimates and their recovered standard errors, with the two matched cohorts treated as independent samples; the formula used and the arithmetic for all four time points are given in S1 Text. Observed event proportions are also reported and are identified as such throughout. Because risk-set counts are not exported, a number-at-risk row could not be added beneath the Kaplan–Meier curve.

All computations were carried out using the TriNetX Analytics Platform, and results with a two-sided *p* value below 0.05 were regarded as statistically significant.

## Results

### Baseline characteristics

In total, 4,839 individuals satisfied the study’s eligibility criteria and had complete data on the matching covariates, of whom 782 had T2DM and 4,057 did not. Following 1:1 propensity-score matching, 755 patients were retained in each group for comparison.

Prior to matching, participants with T2DM were generally older and exhibited greater prevalence of hypertension, hyperlipidemia, ischemic heart disease, chronic kidney disease, and osteoporosis (all *p* < 0.05). Post-matching, baseline characteristics demonstrated satisfactory comparability, with standardized mean differences (SMDs) below 0.10 across all variables (Table 1).

**Table 1:** Patient demographics and comorbidities for Type 2 diabetes mellitus and No diabetes cohorts before and after propensity score matching.

Characteristic	Before propensity matching				After propensity matching			
	T2DM (N = 782)	No diabetes (N = 4,057)	p	SMD	T2DM (N = 755)	No diabetes (N = 755)	p	SMD
Current Age	72 ± 10	68.8 ± 13.1	<0.001	0.291	71.8 ± 10.1	72.4 ± 11.8	0.283	0.055
Age at Index	62.5 ± 11.4	59.1 ± 13.8	<0.001	0.266	62.4 ± 11.5	63.3 ± 12.6	0.191	0.067
Female	392 (50.1%)	2,240 (55.2%)	0.009	0.102	381 (50.5%)	382 (50.6%)	0.959	0.003
Race								
Asian	408 (52.2%)	1,765 (43.5%)	<0.001	0.174	398 (52.7%)	423 (56%)	0.197	0.067
White	269 (34.4%)	1,714 (42.2%)	<0.001	0.162	259 (34.3%)	242 (32.1%)	0.353	0.048
Black	34 (4.3%)	199 (4.9%)	0.505	0.026	34 (4.5%)	28 (3.7%)	0.437	0.04
Other	71 (9.1%)	379 (9.4%)	N/I	N/I	64 (8.5%)	62 (8.2%)	N/I	N/I
Ethnicity								
Hispanic or Latino	25 (3.2%)	99 (2.4%)	0.220	0.046	22 (2.9%)	12 (1.6%)	0.083	0.089
Other	757 (96.8%)	3,958 (97.6%)	N/I	N/I	733 (97.1%)	743 (98.4%)	N/I	N/I
Hypertension	546 (69.8%)	1521 (37.5%)	<0.001	0.685	519 (68.7%)	526 (69.7%)	0.696	0.020
Hyperlipidemia	391 (50%)	897 (22.1%)	<0.001	0.607	364 (48.2%)	346 (45.8%)	0.353	0.048
Osteoarthritis	258 (33%)	844 (20.8%)	<0.001	0.278	239 (31.7%)	249 (33%)	0.582	0.028
Ischemic heart disease	195 (24.9%)	456 (11.2%)	<0.001	0.362	179 (23.7%)	173 (22.9%)	0.715	0.019
Overweight, obesity	144 (18.4%)	310 (7.6%)	<0.001	0.324	126 (16.7%)	130 (17.2%)	0.784	0.014
Nicotine dependence	50 (6.4%)	180 (4.4%)	0.019	0.087	47 (6.2%)	48 (6.4%)	0.916	0.006
Chronic kidney disease	129 (16.5%)	201 (5%)	<0.001	0.379	113 (15%)	105 (13.9%)	0.558	0.030
Osteoporosis	73 (9.3%)	264 (6.5%)	0.004	0.105	71 (9.4%)	76 (10.1%)	0.662	0.022

T2DM, type 2 diabetes mellitus; N/I, no information.

### Early postoperative complications

After matching, the two cohorts showed comparable rates of pneumonia, urinary tract infection, anemia, blood transfusion, and pressure ulcer within 90 days of surgery, and none of these differences reached statistical significance (all p > 0.05; Table 2).

**Table 2:** Early postoperative complications (within 90 days after surgery).

Outcome	T2DM (N = 755)	No diabetes (N = 755)	p	RR (CI 95%)
Pneumonia	15 (2%)	15 (2%)	1	1 (0.492–2.031)
Urinary tract infection	29 (3.8%)	22 (2.9%)	0.319	1.318 (0.764–2.273)
Anemia	25 (3.3%)	35 (4.6%)	0.188	0.714 (0.432–1.181)
Transfusion	20 (2.6%)	13 (1.7%)	0.218	1.538 (0.771–3.07)
Pressure ulcer	57 (7.6%)	65 (8.6%)	0.450	0.877 (0.624–1.233)
SSI	≤ 10	≤ 10	N/I	N/I
Deceased	≤ 10	≤ 10	N/I	N/I

T2DM, type 2 diabetes mellitus; N/I, no information.

Surgical site infection and postoperative mortality were not analyzed because each event occurred in fewer than ten individuals per group, consistent with TriNetX privacy rules that restrict reporting of very small cell counts.

Overall, short-term complication rates after Endo-LIF were similar in patients with and without type 2 diabetes mellitus, with no statistically significant differences observed.

### Healthcare utilization

During the first postoperative year, the rates of emergency department visits and unplanned hospital readmissions were similar between the T2DM and non-diabetes groups, with no significant differences observed in either outcome (both p > 0.05; Table 3). Short-term healthcare-utilization rates after Endo-LIF were therefore similar between the two cohorts, with no statistically significant differences observed.

**Table 3:** Healthcare utilization outcomes (3-, 6- and 12-month follow-up).

Outcome	Time	T2DM (N = 755)	No diabetes (N = 755)	p	RR (CI 95%)
Readmission	3 months	107 (14.2%)	98 (13%)	0.499	1.092 (0.846–1.409)
	6 months	129 (17.1%)	119 (15.8%)	0.487	1.084 (0.863–1.361)
	12 months	188 (24.9%)	166 (22%)	0.181	1.133 (0.943–1.36)
ED visit	3 months	71 (9.4%)	71 (9.4%)	1	1 (0.708–1.413)
	6 months	101 (13.4%)	95 (12.6%)	0.646	1.063 (0.819–1.381)
	12 months	143 (18.9%)	139 (18.4%)	0.792	1.029 (0.833–1.27)

T2DM, type 2 diabetes mellitus; ED, emergency department.

### Code-defined pseudarthrosis

Across all follow-up intervals, code-defined pseudarthrosis was consistently more frequent among patients with T2DM than among those without diabetes. By 6 months the event had been recorded in 73 individuals (9.7%) with T2DM compared with 40 (5.3%) in the non-diabetic group (*p* = 0.001, RR = 1.83, 95% CI 1.26–2.65). At 12 months, the corresponding rates were 10.1% versus 5.7% (*p* = 0.002, RR = 1.77, 95% CI 1.23–2.53), and by 36 months, 11.5% versus 6.2% (*p* < 0.001, RR = 1.85, 95% CI 1.32–2.60) (Table 4). These values are observed proportions and are not adjusted for censoring. The corresponding Kaplan–Meier estimates of cumulative incidence were 9.7% versus 5.3% at 6 months, 10.2% versus 5.8% at 12 months, 11.3% versus 6.1% at 24 months, and 12.0% (95% CI 9.8–14.6) versus 6.4% (95% CI 4.9–8.5) at 36 months (Table 5). The censoring-adjusted estimates were numerically close to the observed proportions, differing by no more than 0.5 percentage points.

**Table 4:** Code-defined pseudarthrosis: observed event proportions (6-, 12- and 36-month follow-up).

Outcome	Time	T2DM (N = 755)	No diabetes (N = 755)	p	RR (CI 95%)
Code-defined pseudarthrosis	6 months	73 (9.7%)	40 (5.3%)	0.001	1.825 (1.258–2.648)
	12 months	76 (10.1%)	43 (5.7%)	0.002	1.767 (1.233–2.534)
	36 months	87 (11.5%)	47 (6.2%)	<0.001	1.851 (1.317–2.601)

T2DM, type 2 diabetes mellitus.

**Table 5:** Kaplan–Meier estimates of the cumulative incidence of code-defined pseudarthrosis (6-, 12-, 24- and 36-month follow-up).

Time	T2DM (N = 755), % (95% CI)	No diabetes (N = 755), % (95% CI)	Absolute risk difference, % (95% CI)
6 months	9.7 (7.8–12.1)	5.3 (3.9–7.2)	4.4 (1.7 to 7.1)
12 months	10.2 (8.2–12.6)	5.8 (4.3–7.7)	4.4 (1.7 to 7.1)
24 months	11.3 (9.2–13.8)	6.1 (4.6–8.1)	5.2 (2.3 to 8.0)
36 months	12.0 (9.8–14.6)	6.4 (4.9–8.5)	5.5 (2.6 to 8.5)

T2DM, type 2 diabetes mellitus; CI, confidence interval. Values are Kaplan–Meier estimates of the cumulative incidence of code-defined pseudarthrosis (ICD-10-CM M96.0), which account for censoring, with 95% confidence intervals derived on the complementary log–log scale; they are therefore not identical to the observed event proportions in Table 4. The absolute risk difference is calculated from the unrounded estimates, treating the two matched cohorts as independent samples, and may therefore differ in the final digit from the difference of the rounded values shown; for the same reason the intervals at 6 and 12 months coincide as displayed although the underlying standard errors differ (S1 Text). The analytics platform does not provide patient-level follow-up times or export the number of patients remaining at risk at each time point, so median follow-up duration and time-specific risk-set counts could not be reported.

**Fig 2:**
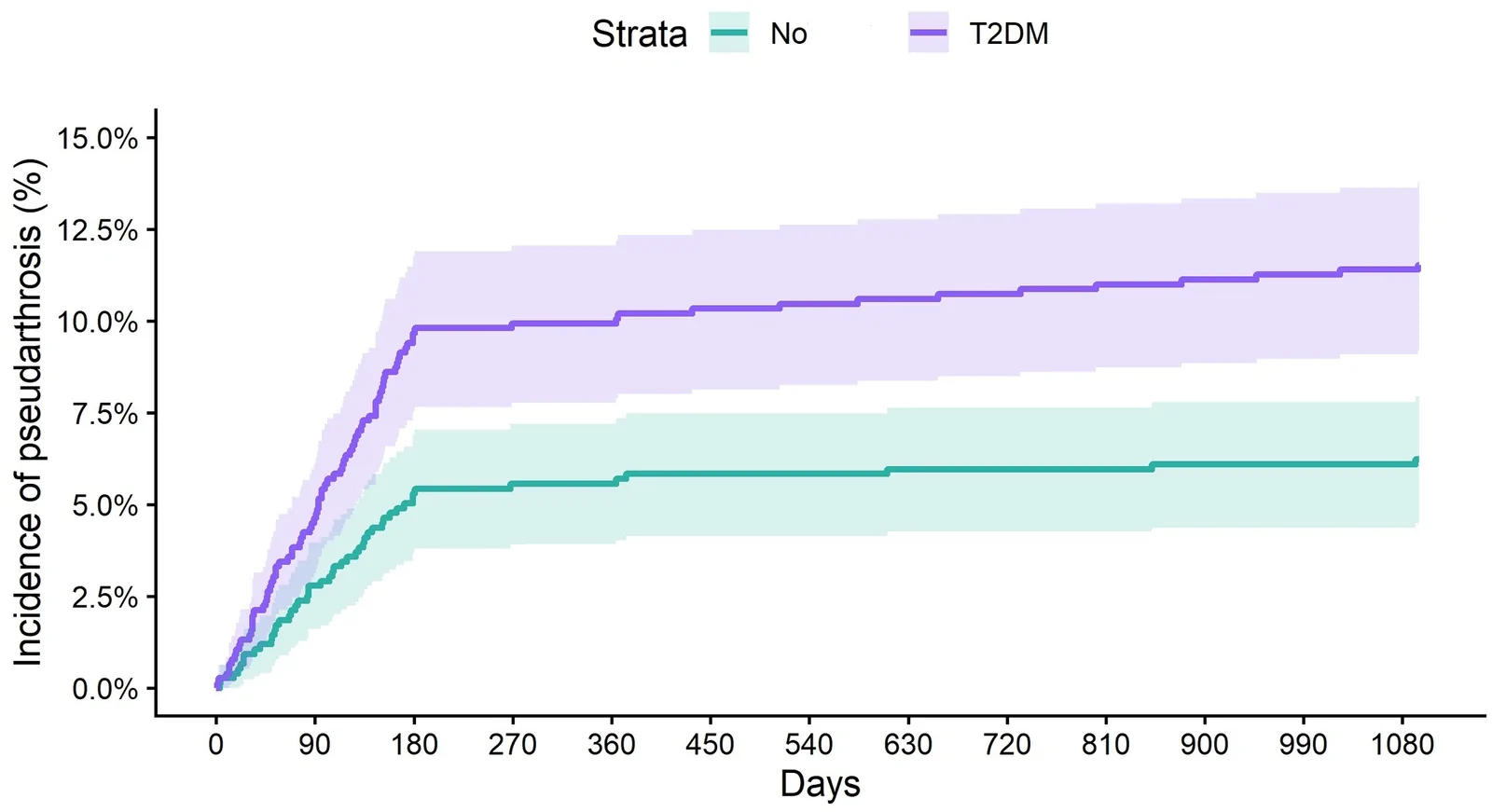
Kaplan–Meier curves showing the cumulative incidence of code-defined pseudarthrosis within 3 years after single-level Endo-LIF. Estimates account for censoring; a number-at-risk row could not be added because the analytics platform does not export the number of patients at risk at each time point.

Kaplan–Meier analysis revealed a significantly higher cumulative incidence of code-defined pseudarthrosis in the diabetic cohort relative to non-diabetic controls (log-rank χ² = 13.09, *p* = 0.0003), and a Cox proportional hazards model yielded a hazard ratio of 1.90 (95% CI 1.33–2.71) (Fig 2). In absolute terms, the censoring-adjusted difference in cumulative incidence at 36 months was 5.5% (12.0% versus 6.4%; 95% CI 2.6% to 8.5%); the difference calculated from observed proportions was 5.3% (95% CI 2.4% to 8.2%). The proportional hazards assumption was satisfied (proportionality test χ² = 0.031, df = 1, p = 0.86), and the complementary log–log curves of the two cohorts were approximately parallel across follow-up (S1 Fig). The analytics platform does not provide patient-level follow-up times and does not export the number of patients remaining at risk at each time point; median follow-up duration and time-specific risk-set counts could therefore not be reported, and a number-at-risk row could not be placed beneath Fig 2.

These findings indicate that type 2 diabetes mellitus was associated with an approximately two-fold higher long-term incidence of code-defined pseudarthrosis following single-level Endo-LIF, despite comparable perioperative complication rates between cohorts.

## Discussion

This multicenter cohort study found that patients with T2DM had a higher incidence of code-defined pseudarthrosis after single-level Endo-LIF. Early postoperative complications and short-term healthcare use were comparable between diabetic and non-diabetic individuals, yet the cumulative incidence of code-defined pseudarthrosis remained higher in the T2DM group throughout three years of follow-up. Similar early outcomes were observed in the two cohorts, whereas the long-term outcome differed. Because the study included no comparator surgical approach, these similar early outcomes cannot be attributed to features of the endoscopic technique itself. In absolute terms the difference was moderate: the Kaplan–Meier cumulative incidence at 36 months was 12.0% versus 6.4%, an absolute risk difference of 5.5% (95% CI 2.6% to 8.5%). The near two-fold relative estimate should therefore be interpreted alongside this absolute difference when counseling patients before surgery. Both measures describe a comparison between exposure groups and should not be read as the expected consequence of an intervention, since T2DM is an observed patient characteristic rather than something assigned.

Our results are consistent with earlier reports in open and minimally invasive lumbar fusion, which have shown impaired bone healing and greater nonunion rates among patients with diabetes [10,28]. Prior studies frequently reported higher perioperative medical complications in diabetic individuals [15,29], a pattern not seen in our matched analysis. Lower tissue disruption has been described for endoscopic approaches [30], but because this study included no comparator surgical approach, the similar early profiles observed in the two cohorts cannot be attributed to features of the endoscopic technique, and they need not extend to the biological processes that determine fusion integrity.

Several metabolic factors associated with chronic hyperglycemia could plausibly contribute to the observed association with code-defined pseudarthrosis. Reduced osteoblast activity, impaired microvascular perfusion, and changes in collagen quality have been implicated in delayed bone remodeling [31,32]. Although these mechanisms were not directly evaluated in our dataset, they align with the persistent association found after adjusting for demographic and clinical variables.

Few studies have examined diabetes-specific outcomes after endoscopic lumbar fusion. By focusing on a population undergoing single-level Endo-LIF as defined by procedure coding, our findings suggest that systemic metabolic health may be associated with long-term fusion outcomes in this population. Because no comparator approach was studied, we cannot say whether this pattern differs from that seen after open or minimally invasive fusion.

Other patient-level factors not captured in claims data may also contribute; for example, lower preoperative muscle strength has been associated with poorer functional outcomes after lumbar interbody fusion [33]. Factors of this kind may be related both to diabetes status and to recovery after surgery, were unavailable for matching, and may therefore contribute to residual confounding.

The timing of the coded events also deserves comment. Most of them appeared within the first six months, whereas radiographic fusion usually takes twelve to twenty-four months to mature. A diagnosis of nonunion at six months is more consistent with delayed union, and because the outcome rests on a diagnosis code, some early entries may reflect transient axial pain or a slow but progressing fusion rather than established nonunion. This supports interpreting the endpoint as code-defined pseudarthrosis rather than radiographically confirmed nonunion. The pattern can be described more precisely. Approximately 84% and 85% of events were recorded within the first six months in the T2DM and non-diabetes cohorts respectively (73 of 87 and 40 of 47). A further 14 and 7 coded events were recorded after six months in the T2DM and non-diabetes cohorts respectively; without the corresponding risk sets, however, these counts cannot support a formal comparison of late incidence. The same pattern governs the absolute difference between the cohorts, which is already 4.4% at six months and rises only to 5.5% by thirty-six months (Table 5). Approximately four-fifths of the three-year absolute difference had therefore already emerged by six months, that is, within the interval in which a coded nonunion is more consistent with delayed union than with established nonunion. The association reported here rests largely on this early coding and should not be read as evidence that T2DM impairs fusion progressively across three years of follow-up. A landmark or sensitivity analysis confined to events first coded after 6 or 12 months would be the appropriate way to separate delayed union from established nonunion, and we regard this as a priority for prospective work in which fusion can be confirmed radiographically.

The coded rate of pressure ulcers (7.6% and 8.6%) was higher than expected after a single-level minimally invasive procedure. It may reflect coding of lesions present before admission, or lesions related to a patient’s care setting such as admission from institutional care, rather than an acute complication of the index operation; notably, the rate was similar in both groups. This pattern illustrates the ascertainment limitation that applies to the primary endpoint: a diagnosis code recorded after the index procedure need not denote a condition arising from it. The same reasoning applies to M96.0 and is a further reason for describing the outcome as code-defined pseudarthrosis throughout.

Several limitations should be acknowledged. First, pseudarthrosis was defined from a diagnosis code rather than confirmed on imaging [34], so the endpoint is code-defined and may misclassify true radiographic nonunion. Second, ICD-10-PCS establishes that the procedure was a single-level percutaneous endoscopic interbody fusion performed from a posterior route, but records nothing further about how it was performed: it does not separate uniportal full-endoscopic from biportal endoscopic technique, nor a posterior interbody fusion from an endoscopic transforaminal variant, and it does not reliably record the graft or implant used. The cohort therefore cannot be characterized at the level of surgical technique, and the findings should be regarded as preliminary. Third, factors that strongly influence fusion, including cage and graft type, BMP use, fixation, endplate preparation, bone quality, surgeon experience, and perioperative bone-active medication such as teriparatide, romosozumab or antiresorptive agents, are not recorded in the database and could not enter the matching model, so part of the observed difference may reflect these rather than diabetes itself. Fourth, HbA1c was available mainly for diagnosis and perioperative values were too sparse to stratify, so the study addresses the presence of T2DM rather than glycemic control or disease duration. Fifth, the study period spanned 2015–2025, over which endoscopic fusion technique, implants, graft strategies and perioperative diabetes management all changed substantially. Year of surgery was not included in the propensity-score model and the distribution of index year across the two cohorts could not be extracted, so secular change in practice remains an unmeasured potential confounder. Sixth, because the outcome is ascertained only through diagnosis coding, differences in follow-up intensity may contribute to the association: patients with T2DM may have more frequent healthcare encounters and therefore more opportunities to receive an M96.0 code, independently of true fusion status. In addition, the index window extended to January 1, 2025 while the data were accessed on July 7, 2025, so patients treated late in the period could not contribute a full three years of potential follow-up; Kaplan–Meier estimates are reported for this reason, but the number of patients remaining at risk at each time point could not be exported from the platform. Finally, the platform compares matched cohorts as independent samples and does not permit patient-level export, so the paired structure could not be modeled directly, and because the network is continuously updated the exact cohort sizes reported here would not be reproduced by an identical query run at a later date. Prospective studies with imaging-confirmed fusion and recorded surgical and metabolic detail are needed to clarify how specific features of diabetes relate to fusion outcomes.

Despite these limitations, the study has several strengths. The large multicenter dataset improves generalizability, and standardized data extraction helps reduce inter-institutional variability. Propensity score matching reduced baseline imbalance, and limiting the cohort to single-level procedures reduced heterogeneity in the number of levels fused, although the surgical technique itself could not be characterized. Taken together, these findings indicate that although perioperative outcomes were similar between the two cohorts, T2DM remained associated with the long-term outcome.

## Conclusion

In this database cohort, T2DM was associated with a higher rate of code-defined pseudarthrosis after single-level Endo-LIF, while short-term complications and healthcare use were similar in the two groups. Because the outcome was defined by a diagnosis code rather than imaging, the operation could not be characterized by technique, and important surgical variables were unavailable, these preliminary findings should be interpreted as an association and do not establish a causal effect of T2DM on radiographic nonunion. They are consistent with, but do not demonstrate, a contribution of systemic metabolic factors to long-term fusion, and require confirmation in prospective studies that verify fusion on imaging and record technique and graft detail.

## Supporting information

S1 Fig Assessment of the proportional hazards assumption. Complementary log–log plot of the cumulative hazard of code-defined pseudarthrosis against follow-up time (days, log scale) for the T2DM and non-diabetes cohorts. The two curves are approximately parallel across follow-up. This is consistent with the formal proportionality test based on scaled Schoenfeld residuals (χ² = 0.031, df = 1, p = 0.86) and supports the proportional hazards assumption underlying the reported hazard ratio.
(TIFF)

S1 Text Derivation of the standard errors and absolute risk differences from the reported Kaplan–Meier confidence limits. Sets out the relationship between a complementary log–log confidence interval and the standard error of the survival estimate, the recovery of that standard error from the limits reported by the analytics platform, and the resulting absolute risk differences and confidence intervals at 6, 12, 24 and 36 months.
(DOCX)

## Abbreviations

T2DM: Type 2 diabetes mellitus
Endo-LIF: Endoscopic lumbar interbody fusion
ICD-10-CM/PCS: International Classification of Diseases, 10th Revision, Clinical Modification and Procedure Coding System
HIPAA: Health Insurance Portability and Accountability Act
ADA: American Diabetes Association
PSM: Propensity-score matching
SMD: standardized mean difference
N/I: No information
SSI: Surgical site infection
RR: Risk ratio
HR: Hazard ratio
CI: Confidence interval
