# Supplementary material for: Association of type 2 diabetes mellitus with postoperative outcomes following single-level endoscopic lumbar interbody fusion through a posterior approach: A global propensity score–matched cohort study
Source: PLoS One. 2026 Sep 18;21(9):e0358422. doi: 10.1371/journal.pone.0358422 (PMC13588496; doi:10.1371/journal.pone.0358422)
Supplement: Supplementary file 1 [file pone.0358422.s002.docx]

**S1 Text. Derivation of the standard errors and absolute risk differences from the reported Kaplan–Meier confidence limits.**

Association of type 2 diabetes mellitus with postoperative outcomes following single-level endoscopic lumbar interbody fusion through a posterior approach: a global propensity score–matched cohort study

**1. Source of the values and why the standard errors were recovered**

The TriNetX Analytics Platform exports the Kaplan–Meier graph data as a step table giving, for each cohort at every step, the survival probability together with its 95% confidence limits. The limits are constructed on the complementary log–log scale. The export does not contain the number of patients remaining at risk or the number of events at each event time, so the Greenwood variance cannot be evaluated directly from the risk sets. The standard error of each estimate was therefore recovered from the exported confidence limits, which are a monotone transformation of that standard error. Every quantity below was computed from the unrounded values in that export; none was read from the rounded figures displayed in Table 5.

**2. Recovering the standard error from a complementary log–log interval**

Let S(t) be the survival function and F(t) = 1 – S(t) the cumulative incidence. Writing σ for the standard error of log[–log S(t)], the complementary log–log interval for S(t) is

S_lower = S^exp(+1.96σ)	and	S_upper = S^exp(–1.96σ)

Taking logarithms of either limit and solving for σ gives

σ = – ln[ ln(S_upper) / ln(S) ] / 1.96	(from the upper limit)

σ = + ln[ ln(S_lower) / ln(S) ] / 1.96	(from the lower limit)

By the delta method the standard error of the survival estimate, and hence of the cumulative incidence, is

SE[F(t)] = SE[S(t)] = σ × S(t) × |ln S(t)|

The two limits provide an internal check: if the interval is indeed constructed on the complementary log–log scale, the two expressions for σ must agree exactly. Across the eight estimates used here they agree to within 6e-16, that is to the limit of floating-point precision, confirming both the scale on which the platform constructs its intervals and the validity of the recovery.

**3. Absolute risk difference**

The two matched cohorts are analysed by the platform as independent samples throughout, as described in the Statistical Analysis section. The absolute risk difference at time t and its 95% confidence interval are therefore

ARD = F₁(t) – F₂(t)

SE(ARD) = √[ SE(F₁)² + SE(F₂)² ]

95% CI = ARD ± 1.96 × SE(ARD)

**4. Recovered standard errors**

Cumulative incidence and standard errors are percentages; σ is dimensionless. Time points correspond to 180, 365, 730 and 1096 days after the index procedure.

| **Time (months)** | **Cohort** | **Cumulative incidence % (95% CI)** | **σ** | **SE** | **SE(ARD)** |
| --- | --- | --- | --- | --- | --- |
| 6 | T2DM | 9.747 (7.828–12.104) | 0.11712 | 1.0840 | 1.3606 |
|  | No diabetes | 5.344 (3.948–7.215) | 0.15816 | 0.8223 |  |
| 12 | T2DM | 10.179 (8.214–12.580) | 0.11483 | 1.1071 | 1.3994 |
|  | No diabetes | 5.779 (4.317–7.714) | 0.15261 | 0.8559 |  |
| 24 | T2DM | 11.271 (9.188–13.790) | 0.11010 | 1.1682 | 1.4631 |
|  | No diabetes | 6.090 (4.581–8.074) | 0.14930 | 0.8809 |  |
| 36 | T2DM | 11.981 (9.814–14.586) | 0.10787 | 1.2116 | 1.5171 |
|  | No diabetes | 6.447 (4.878–8.497) | 0.14646 | 0.9131 |  |

**5. Absolute risk differences**

The unrounded limits are shown alongside the values as they appear in Table 5.

| **Time (months)** | **ARD** | **1.96 × SE(ARD)** | **Unrounded 95% CI** | **As reported in Table 5** |
| --- | --- | --- | --- | --- |
| 6 | 4.4024 | 2.6667 | 1.7356 to 7.0691 | 4.4 (1.7 to 7.1) |
| 12 | 4.4000 | 2.7428 | 1.6572 to 7.1429 | 4.4 (1.7 to 7.1) |
| 24 | 5.1816 | 2.8677 | 2.3139 to 8.0493 | 5.2 (2.3 to 8.0) |
| 36 | 5.5338 | 2.9736 | 2.5602 to 8.5074 | 5.5 (2.6 to 8.5) |

Two features of this table are worth stating explicitly, since both could otherwise be mistaken for transcription errors. First, at 36 months the difference of the rounded cohort estimates is 12.0 – 6.4 = 5.6, whereas the difference itself is 5.5338 and rounds to 5.5. Second, the confidence intervals at 6 and 12 months are identical as displayed although the underlying standard errors differ (1.3606 and 1.3994): the unrounded limits are 1.7356 to 7.0691 and 1.6572 to 7.1429, which coincide only after rounding to one decimal place.

**6. Worked example (36 months, T2DM cohort)**

The exported survival probability is S = 0.8801943764, so the cumulative incidence is 11.9806% and ln S = -0.127613. The exported confidence limits for S are 0.8541431475 and 0.9018618714, with ln S_lower = -0.157656 and ln S_upper = -0.103294.

From the upper limit, σ = – ln(-0.103294 / -0.127613) / 1.96 = 0.107867; from the lower limit, σ = ln(-0.157656 / -0.127613) / 1.96 = 0.107867. The two agree, as required.

Hence SE = 0.107867 × 0.880194 × 0.127613 = 0.012116, that is 1.2116%. The corresponding value for the non-diabetes cohort is 0.9131%, so SE(ARD) = √(1.2116² + 0.9131²) = 1.5171% and 1.96 × SE(ARD) = 2.9736%. With ARD = 5.5338%, the 95% confidence interval is 2.5602% to 8.5074%, reported as 5.5% (95% CI 2.6% to 8.5%).
